# DNABERT-S: LEARNING SPECIES-AWARE DNA EMBEDDING WITH GENOME FOUNDATION MODELS

**Published:** 2024-02-15

**Authors:** Zhihan Zhou, Weimin Wu, Harrison Ho, Jiayi Wang, Lizhen Shi, Ramana V Davuluri, Zhong Wang, Han Liu

**Affiliations:** † Department of Computer Science, Northwestern University, Evanston, IL, USA; ‡ School of Natural Sciences, University of California at Merced, Merced, CA, USA; § Department of Energy Joint Genome Institute, Lawrence Berkeley National Laboratory, Berkeley, CA, USA; ¶ Department of Statistics and Data Science, Northwestern University, Evanston, IL, USA; ∥ Department of Biomedical Informatics, Stony Brook University, Stony Brook, NY, USA; ** Environmental Genomics and Systems Biology Division, Lawrence Berkeley National Laboratory, Berkeley, CA, USA

## Abstract

Effective DNA embedding remains crucial in genomic analysis, particularly in scenarios lacking labeled data for model fine-tuning, despite the significant advancements in genome foundation models. A prime example is metagenomics binning, a critical process in microbiome research that aims to group DNA sequences by their species from a complex mixture of DNA sequences derived from potentially thousands of distinct, often uncharacterized species. To fill the lack of effective DNA embedding models, we introduce DNABERT-S, a genome foundation model that specializes in creating species-aware DNA embeddings. To encourage effective embeddings to error-prone long-read DNA sequences, we introduce Manifold Instance Mixup (MI-Mix), a contrastive objective that mixes the hidden representations of DNA sequences at randomly selected layers and trains the model to recognize and differentiate these mixed proportions at the output layer. We further enhance it with the proposed Curriculum Contrastive Learning (C^2^LR) strategy. Empirical results on 18 diverse datasets showed DNABERT-S’s remarkable performance. It outperforms the top baseline’s performance in 10-shot species classification with just a 2-shot training while doubling the Adjusted Rand Index (ARI) in species clustering and substantially increasing the number of correctly identified species in metagenomics binning. The code, data, and pre-trained model are publicly available at https://github.com/Zhihan1996/DNABERT_S.

## 1 Introduction

The introduction of genome foundation models, including DNABERTs and HyenaDNA [Dalla-Torre et al., 2023; Ji et al., 2021; Nguyen et al., 2023; Zhou et al., 2023], marks a transformative era in genomic analysis. These models, enriched with extensive domain knowledge through large-scale pre-training, have demonstrated impressive capabilities in various genome analysis tasks, especially where labeled data is available. However, a critical challenge persists: the lack of labeled data in key applications, such as metagenomics binning.

Metagenomics binning [Kang et al., 2015, 2019; Lamurias et al., 2023; Meyer et al., 2022; Nissen et al., 2021] aims to group DNA sequences from a complex mixture of multiple organisms, typically in the absence of prior labeling, which is essential for supervised fine-tuning. In this context, high-quality and discriminative DNA embeddings become an indispensable tool. Similarly, other important problems such as nucleosome positioning [Han et al., 2022] and DNA sequence comparison [Ren et al., 2022], and various biological analysis problems involving multi-modal data (e.g., gene, protein, pathway, etc.) [Ma et al., 2023] where direct model fine-tuning is not viable, also benefit substantially from effective DNA embeddings.

Despite the critical role of DNA embeddings in genomic research, the current landscape reveals a notable shortfall in effective methods for their development. Current approaches primarily achieve DNA embeddings via three methods: 1) Descriptive textual features [Kang et al., 2015, 2019; Nissen et al., 2021], 2) Pre-trained Kmer embeddings [Han et al., 2022; Ng, 2017; Ren et al., 2022], and 3) Genome foundation models [Ji et al., 2021; Nguyen et al., 2023; Zhou et al., 2023]. The first two methods, while straightforward, fail to grasp complex semantic relationships. Moreover, existing genome foundation models, predominantly pre-trained with language modeling objectives [Devlin et al., 2018; Radford et al., 2019], often fail to yield representative embeddings [Gao et al., 2021; Reimers and Gurevych, 2019]. Our empirical analysis reveals that, in many scenarios, existing genome foundation models even underperform descriptive textual features, as detailed in Table 1.

**Figure 1:**
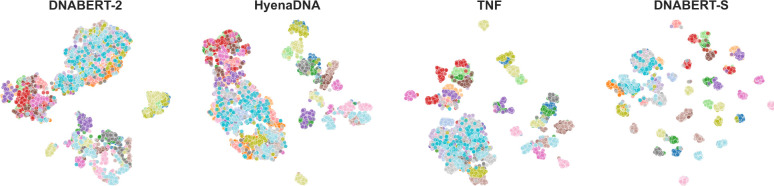
TSNE visualization of the DNA embeddings generated by different methods on a CAMI2 [Meyer et al., 2022] dataset with 50 different species. Each point represents an individual DNA sequence, with the color coding indicating the species affiliation. Notably, DNABERT-S demonstrates a pronounced ability to cluster and segregate different species within the embedding space.

To address this challenge, we introduce DNABERT-S, a specialized genome foundation model tailored for generating species-aware DNA embeddings. As depicted in Figure 1, DNABERT-S distinguishes itself from other methods by its ability to effectively cluster and separate different species within the embedding space. This enhanced performance stems from the proposed Manifold Instance Mixup (MI-Mix) loss and Curriculum Contrastive Learning (C^2^LR) strategy. Contrastive learning enables the model to discern between similar and dissimilar DNA sequences, and curriculum learning incrementally presents more challenging training samples, fostering better learning and generalization. The training of DNABERT-S includes two phases: 1) differentiate similar and dissimilar sequences; 2) form more challenging contrastive anchors and continue the differentiation. In the first phase, we adopt a Weighted SimCLR Chen et al. [2020]; Zhou et al. [2022] training objective to encourage the model to group similar DNA sequences and separate dissimilar DNA sequences. In the second phase, we introduce Manifold Instance Mixup (MI-Mix) which mixes anchor instances at a randomly selected layer to create more challenging anchors for contrastive training.

To evaluate DNABERT-S against existing methods, we compiled a comprehensive benchmark that included more than 1,000 species, reflecting the diversity of natural microbial communities. This benchmark includes complex datasets from CAMI2 [Meyer et al., 2022], a leading metagenomics binning benchmark, and extensive reference genomes from Genbank [Benson et al., 2012]

We evaluate the methods on three types of tasks: metagenomics binning, clustering, and classification, from entirely unsupervised problems to few-shot learning. Experimental results indicate the remarkable performance of DNABERT-S over all the existing methods. Compared to the strongest existing method, DNABERT-S doubles its performance in the clustering task and achieves better performance with only 20% of labeled data in the classification task (e.g., 2-shot v.s. 10-shot). Besides, in metagenomics binning, we show DNABERT-S is able to recover over 40% and 80% of species with an F1 score of over 0.5 from respectively from synthetics and more realistic datasets, which is also one time more than the strongest baseline.

Our contribution can be summarized as follows: 1) For the first time, we demonstrate the superiority of genome foundation models in learning effective DNA embeddings, opening new avenues for tackling a wide range of genomic research challenges; 2) We introduce DNABERT-S, a genome foundation model distinctly outperforming existing methods in learning DNA embeddings; 3) We introduce the Curriculum Contrastive Learning (C^2^LR) strategy with the Manifold Instance Mixup (MI-Mix) loss, which effectively facilitate DNA embedding learning; 4) We construct a large-scale evaluation benchmark for DNA embedding.

## 2 Background and Related Work

This study delves into the problem of species-aware DNA embedding, aiming to create a model that maps each DNA sequence as a fixed-size numerical vector in a vector space, where sequences from distinct species are naturally clustered and segregated. A DNA sequence is essentially a string composed of four unique characters: A, T, C, and G.

Existing works highly rely on descriptive textual features [Kang et al., 2015, 2019; Nissen et al., 2021] and pre-trained K-mer embeddings [Han et al., 2022; Ng, 2017; Ren et al., 2022] to compute DNA embeddings. A representative descriptive textual feature is Tetra-Nucleotide Frequency (TNF), a 256-dimensional vector where each position represents the frequency of each unique 4-mer (e.g., TTCA, AACG) in the input DNA sequence. Despite its simplicity and effectiveness, this method is limited since it is not trainable to better fit downstream applications. Besides, our empirical analysis also suggests that a naive trainable model based on TNF, such as a Variational AutoEnoder [Kingma and Welling, 2013] with TNF as input, results in worse embeddings compared to TNF. With the success of Word2Vec [Mikolov et al., 2013], pre-trained Kmer embeddings have gained popularity in computing DNA embeddings for various applications [Han et al., 2022; Ng, 2017; Ren et al., 2022]. However, the emergence of deep learning advancements such as ELMo and BERT [Devlin et al., 2018; Peters et al., 2018] highlights the limitations of static word embeddings compared to contextual embeddings produced by foundation models. Recently, Genome Foundation Models such as DNABERT-2 and HyenaDNA have demonstrated their prowess in genome analysis [Dalla-Torre et al., 2023; Ji et al., 2021; Nguyen et al., 2023; Zhou et al., 2023]. However, in the absence of task-specific data, these models struggle, largely due to the mismatch between their language-modeling training objectives and the goal of producing distinctive embeddings [Li et al., 2020].

In response, we turn to contrastive learning [Chen et al., 2020; Gao et al., 2021; Lee et al., 2020; Reimers and Gurevych, 2019] with genome foundation model and introduce the curriculum contrastive learning (C^2^LR) strategy with the Manifold Instance Mixup (MI-Mix) training objective.

## 3 Model

The proposed Curriculum Contrastive Learning (C^2^LR) splits the training process into two phases, gradually creating more challenging anchors. In phase I, we apply an effective contrastive learning method named Weighted SimCLR based on SimCLR and Hard-Negative sampling strategy (Sec. 3.1). In phase II, we propose the Manifold Instance Mixup method which creates more challenging anchors by mixing intermediate hidden states of inputs in a randomly selected hidden layer of the model (Sec. 3.2). Implementation details of DNABERT-S are presented in Sec. 3.3.

### Notation:

Let $x_{i}$ define an input sample. Consider a batch ${\left\{\left(x_{i},x_{i^{+}}\right)\right\}}_{i=1}^{B}$, where B is the batch size and $\left(x_{i},x_{i^{+}}\right)$ represents a pair of samples that we consider to be similar (a.k.a., positive pair). In our setting, a positive pair $\left(x_{i},x_{i^{+}}\right)$ represents two non-overlapping DNA sequences from the same genome. Let f(⋅) define the embedding model, which takes $x_{i}$ as input and computes fixed-size embedding $f\left(x_{i}\right)$.

### 3.1 Weighted SimCLR

SimCLR [Chen et al., 2020] is a simple and effective framework for contrastive learning. For an anchor $x_{i}$ in batch ${\left\{\left(x_{i},x_{i^{+}}\right)\right\}}_{i=1}^{B}$, SimCLR treats all the other 2B-2 samples in the same batch as negative samples. It encourages the model to increase the anchor’s similarity with its positive sample $x_{i^{+}}$ and reduces its similarity with the negative samples. It treats all negative samples equally. However, recent works [Zhang et al. 2021] have suggested that hard negatives that are closer to the anchor in the representation space offer more informative learning contrasts. Therefore, Weighted SimCLR [Zhang et al. 2021] gives higher weights to negative samples that are closer to the anchor. To align with subsequent sections, we introduce the virtual labels. The label for $\left(x_{i},x_{i^{+}}\right)$ is $v_{i}\in \{0,1{\}}^{B}$, where $v_{i,i}=1$ indicates positive samples, and $v_{i,j\neq i}=0$ indicates negative samples. The Weighted SimCLR loss for $x_{i}$ is defined as:

(1) $$\ell \left(f\left(x_{i}\right),v_{i}\right)=-\sum_{n=1}^{B}v_{i,n}\log\frac{\exp\left(s\left(f\left(x_{i}\right),f\left(x_{n^{+}}\right)\right)/\tau \right)}{\sum_{j\neq i}\alpha_{ij}\exp\left(s\left(f\left(x_{i}\right),f\left(x_{j}\right)\right)/\tau \right)},$$

where τ denotes the temperature and s(⋅,⋅) denotes the cosine similarity between two inputs. Weights $\alpha_{ij}$ denotes the relative importance of $x_{j}$ for optimizing the contrastive loss of anchor $x_{i}$ among all the 2B-2 negative samples. A negative sample that is closer to the anchor receives a higher weight. We set $\alpha_{ii^{+}}=1$ and compute $\alpha_{ij}$ as:

$$\alpha_{ij}=\frac{exp\left(s\left(f\left(x_{i}\right),f\left(x_{j}\right)\right)/\tau \right)}{\frac{1}{2B-2}\sum_{k\neq i,i^{+}}exp\left(s\left(f\left(x_{i}\right),f\left(x_{k}\right)\right)/\tau \right)}.$$

For each positive pair $\left(x_{i},x_{i^{+}}\right)$, Weighted SimCLR respectively takes $x_{i}$ and $x_{i^{+}}$ as the contrastive anchors to calculate the contrastive loss. It defines the loss $\ell \left(f\left(x_{i^{+}}\right),v_{i}\right)$ for $x_{i^{+}}$ by exchanging the roles of instances ${\left\{x_{i}\right\}}_{i=1}^{B}$ and ${\left\{x_{i^{+}}\right\}}_{i=1}^{B}$ in Eq. (1) respectively. Therefore, the Weighted SimCLR loss on the entire batch is defined as:

(2) $$ℒ=\frac{1}{2B}\sum_{i=1}^{B}\left(\ell \left(f\left(x_{i}\right),v_{i}\right)+\ell \left(f\left(x_{i^{+}}\right),v_{i}\right)\right).$$

### 3.2 Curriculum Contrastive Learning (C^2^LR)

In this part, we introduce our curriculum contrastive learning (C^2^LR) method. Curriculum learning is an effective training method that first presents easy training batches and then progresses to more challenging ones [Hacohen and Weinshall, 2019]. Recent studies have successfully applied this technique to both positive pairs [Roy and Etemad, 2023; Ye et al., 2021] and negative pairs [Chu et al., 2021] in contrastive learning. We take this approach a step further by applying it to contrastive anchors, effectively using it for both types of pairs at the same time.

**Figure 2:**
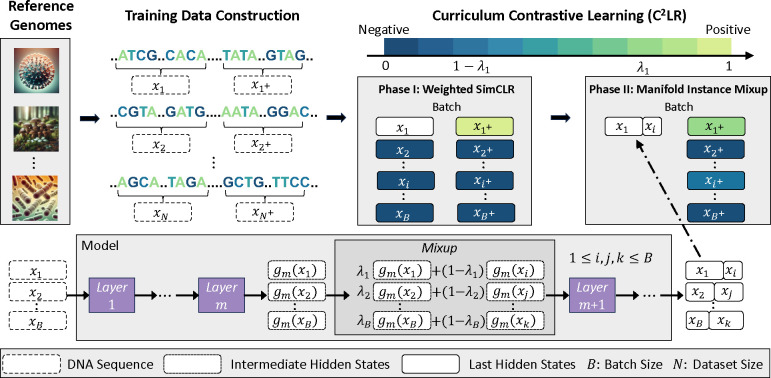
Overview of DNABERT-S’s training process. We construct training data from massive reference genomes and train DNABERT-S with the proposed Curriculum Contrastive Learning (C^2^LR) strategy that progressively provides more challenging contrastive anchors to the model in two different phases. We propose the Manifold Instance Mixup (MI-Mix) objective that mixes the intermediate hidden states of different inputs to construct the mixed and challenging contrastive anchor.

As shown in Figure 2, our C^2^LR method includes two training phases, with anchors becoming progressively more challenging. In phase I, we use the Weighted SimCLR introduced in Sec. 3.1. In phase II, we propose the Manifold Instance Mixup (MI-Mix) method to mix up anchor instances in a random hidden layer, motivated by the instance mixup (i-Mix) method [Lee et al., 2020].

The i-Mix method mixes anchors at the input layer to create more challenging positive and negative pairs. It only uses the samples from ${\left\{x_{i}\right\}}_{i=1}^{B}$ as anchors and only considers the positive and negative samples from ${\left\{x_{i^{+}}\right\}}_{i=1}^{B}$. Otherwise, it nearly doubles the memory or training time compared to the Weighted SimCLR method in Sec. 3.1 (see Appendix A for details). To perform mixup within the anchor space, i-Mix first shuffles ${\left\{\left(x_{i},v_{i}\right)\right\}}_{i=1}^{B}$ to generate ${\left\{\left({\overset{ˆ}{x}}_{i},{\overset{ˆ}{v}}_{i}\right)\right\}}_{i=1}^{B}$, i.e. a random permutation of ${\left\{\left(x_{i},v_{i}\right)\right\}}_{i=1}^{B}$. Then for each anchor $\left(x_{i},v_{i}\right)$, i-Mix mixes it with $\left({\overset{ˆ}{x}}_{i},{\overset{ˆ}{v}}_{i}\right)$ through weighted sum. The mixing weight $\lambda_{i}$ is drawn from Beta(α,α), where α is a hyperparameter.

Despite i-Mix’s effectiveness on continuous data such as images and speeches, directly mixing DNA sequences may avoid biological plausibility. Thus, we proposed to instead mix hidden representations of DNA sequences at a deeper layer, which essentially combines more abstract, higher-level features of the sequences. We call it Manifold Instance Mixup, inspired by Verma et al. [2019]. Concretely, we denote the model f(⋅) as $f(x)=f_{m}\left(g_{m}(x)\right)$. Here, $g_{m}(\cdot )$ maps input data to the intermediate hidden states at layer m, and $f_{m}(\cdot )$ maps these intermediate hidden states to the output f(x).

The Manifold Instance Mixup includes four steps. First, we uniformly select a random layer m from a set of eligible layers 𝒮 in the model, like one of the encoder layers in DNABERT-S. Second, for a batch of anchors ${\left\{\left(x_{i},v_{i}\right)\right\}}_{i=1}^{B}$, we process them up to layer m, resulting in a batch of intermediate hidden states ${\left\{\left(g_{m}\left(x_{i}\right),v_{i}\right)\right\}}_{i=1}^{B}$. Third, we shuffle ${\left\{\left(g_{m}\left(x_{i}\right),v_{i}\right)\right\}}_{i=1}^{B}$ to get ${\left\{\left(g_{m}\left({\overset{ˆ}{x}}_{i}\right),{\overset{ˆ}{v}}_{i}\right)\right\}}_{i=1}^{B}$ and mix them up. This produces the mixed hidden states $h_{i}^{m}$ for each $\left(g_{m}\left(x_{i}\right),v_{i}\right)$, where $h_{i}^{m}=\lambda_{i}g_{m}\left(x_{i}\right)+\left(1-\lambda_{i}\right)g_{m}\left({\overset{ˆ}{x}}_{i}\right)$, and $v_{i}^{mix}=\lambda_{i}v_{i}+\left(1-\lambda_{i}\right){\overset{ˆ}{v}}_{i}$. Fourth, we feed ${\left\{h_{i}^{m}\right\}}_{i=1}^{B}$ through the remaining layers to get the last hidden states ${\left\{f_{m}\left(h_{i}^{m}\right)\right\}}_{i=1}^{B}$. Loss on i-th anchor $\left(x_{i},v_{i}\right)$ is defined as:

$$\hat{\ell }\left(f_{m}\left(h_{i}^{m}\right),v_{i}^{mix}\right)=-\sum_{n=1}^{B}v_{i,n}^{mix}log\frac{exp\left(s\left(f_{m}\left(h_{i}^{m}\right),f\left(x_{n^{+}}\right)\right)/\tau \right)}{\sum_{j=1}^{B}\alpha_{ij^{+}}exp\left(s\left(f_{m}\left(h_{i}^{m}\right),f\left(x_{j^{+}}\right)\right)/\tau \right)},$$

where weights $\alpha_{ii^{+}}=1$ and $\alpha_{ij^{+}}$ is computed as:

$$\alpha_{ij^{+}}=\frac{exp\left(s\left(f_{m}\left(h_{i}^{m}\right),f\left(x_{j^{+}}\right)\right)/\tau \right)}{\frac{1}{B-1}\sum_{k=1,k\neq i}^{B}exp\left(s\left(f_{m}\left(h_{i}^{m}\right),f\left(x_{k^{+}}\right)\right)/\tau \right)}.$$

The Manifold Instance Mixup loss is defined as follows:

(3) $$\hat{ℒ}=\frac{1}{B}\sum_{i=1}^{B}\hat{\ell }\left(f_{m}\left(h_{i}^{m}\right),v_{i}^{mix}\right).$$

### 3.3 Implementation

In the C^2^LR method, we set temperature τ as 0.05 and hyperparameter α as 1.0. We train the model for one epoch in phase I using loss Eq. (2) and for two epochs in phase II using loss Eq. (3). We use mean pooling of the last hidden states of all the tokens as the DNA embedding. We employ the Adam optimizer [Kingma and Ba, 2014], with a learning rate of 3e-6 and batch size of 48. We save the model every 10000 training steps and select the best one based on the validation loss in the validation dataset. We use the pre-trained DNABERT-2 [Zhou et al., 2023] as the starting point of contrastive training. We also conduct parallel experiments with HyenaDNA [Nguyen et al., 2023]. In Appendix C.1, we show that DNABERT-2 outperforms HyenaDNA after the same contrastive training. The entire training process of DNABERT-S takes approximately 48 hours on 8 NVIDIA A100 80GB GPUs.

## 4 Data

In this section, we introduce the dataset we used for DNABERT-S training and evaluation.

### 4.1 Training

Each training sample of DNABERT-S is a pair of non-overlapping DNA sequences extracted from the same genome. The dataset is constructed with the reference genomes from GenBank [Benson et al., 2012]. We obtained 47923 pairs from 17636 viral genomes, 1 million pairs from 5011 fungi genomes, and 1 million pairs from 6402 bacteria genomes. We randomly selected 2 million pairs from the entire 2047923 pairs of DNA sequences to construct the training data. The rest pairs are treated as validation data. All the DNA sequences are 10000 bp in length.

### 4.2 Evaluation

Our evaluation spans on the Critical Assessment of Metagenome Interpretation (CAMI) II [Meyer et al., 2022] challenge benchmark and 4 synthetic datasets. CAMI2 is one of the most comprehensive and rigorous benchmarks for metagenomics research. The datasets in CAMI2 are designed to mimic realistic microbiome environments and include a vast array of both new and known genomes, as well as plasmids and viruses. It aligns our study with real-world ecological and biological scenarios, providing a robust and contextually relevant evaluation for the DNA embedding models. We utilize 7 datasets of long-read contigs respectively from the Marine and Plant-associated environments, where each dataset consists of 150k-200k DNA sequences belonging to about 100 – 750 different species sampled from 1680 microbial genomes and 599 circular elements. We also create 4 Synthetic datasets by randomly extracting DNA sequences from fungi and viral reference genomes that **do not overlap** with our training data. Table 3 shows the statistics of the datasets we used for evaluation.

To comprehensively understand the impact of DNA embedding in various scenarios, we conduct three types of tasks: 1) species clustering given the number of species, 2) species classification with different numbers of labeled samples, and 3) metagenomics binning with an unknown number of species. The first two tasks evaluate the models in a more standard yet unrealistic setting, while the third task is a realistic problem. Since the CAMI2 datasets are highly imbalanced, for the clustering and classification tasks, we filtered the datasets to eliminate species with fewer than 100 sequences and only kept 100 sequences for each species, resulting in a set of perfectly balanced datasets. For the metagenomics binning problem, to mimic real-world scenarios, where the number of existing samples is unknown, we do not balance the data. Instead, following Kang et al. [2015], we only keep DNA sequences longer than 2500bp and filter out species with fewer than 10 sequences.

## 5 Experiments

In this section, we present experimental design and empirical results. We introduce baselines in Sec. 5.1 and respectively present the results of clustering in Sec. 5.2, classification in Sec. 5.3, and metagenomics binning in Sec. 5.4. In Sec. 5.5, we present ablation studies on curriculum learning and the proposed Manifold Instance Mixup training objective. We also provide empirical analysis on varying backbone models, different input lengths, reduced feature dimensions, scenarios with abundant training data, and various other types of tasks. Due to space limitations, we present them in Appendix C. For all tasks, we perform 5 independent runs with different random seeds for each model and report the averaged results.

### 5.1 Baselines

We compare our model with four lines of work to examine its effectiveness in generating DNA embedding.

**TNF, TNF-K,** and **TNF-VAE** are the most widely used DNA embedding methods in metagenomics binning tools [Kang et al., 2015, 2019; Nissen et al., 2021]. **TNF** represents Tetra-Nucleotide Frequency, which uses the appearance frequency of each unique 4-mer (4^4^ = 256 in total) in a DNA sequence as its embedding. **TNF-K** [Nissen et al., 2021] reduces TNF to 103-dimension with a linear kernel, which utilizes DNA characteristics to reduce the correlations among different dimensions of the original TNF feature. **TNF-VAE** trains a Variational Autoencoder [Kingma and Welling, 2013] using TNF as input to extract features.

**DNA2Vec** [Ng, 2017] learns pre-trained K-mer embedding inspired by Mikolov et al. [2013]. We set K=4 to make it directly comparable with TNF and use the average of the 4-mer embeddings as the DNA embedding.

**DNABERT-2** [Zhou et al., 2023], **HyenaDNA** [Nguyen et al., 2023], and **NT (Nucleotide Transformer)** [Dalla-Torre et al., 2023] are representative genome foundation models. We use the average of the last hidden states as the DNA embedding. For evaluations, we utilized the respective pre-trained models from Huggingface ModelHub, specifically *zhihan1996/DNABERT-2–117M*, *LongSafari/hyenadna-medium-450k-seqlen-hf*, and *InstaDeepAI/nucleotide-transformer-v2–100m-multi-species*.

**DNA-Mutate, DNA-Dropout,** and **DNA-Double** are variants of DNABERT-S, with the same hyperparameters and starting checkpoint but different positive pair construction strategies in contrastive training. **DNA-Mutate** views the same DNA sequence before and after random mutation (i.e., swap and delete 5% of nucleotides) as a positive pair. **DNA-Dropout** is inspired by Gao et al. [2021], which passes the same DNA sequence through the embedding model (with a dropout rate as 0.1) twice and views the two distinct embeddings as a positive pair. **DNA-Double** views a DNA sequence and its complementary (e.g., AATTC v.s. TTAAG) as a positive pair.

### 5.2 Clustering

In this task, we evaluate the embedding quality by how well a standard clustering algorithm can distinguish and cluster different species based on the embedding. To reduce the effects of other factors, we assume the number of species is known in this task. For each dataset, we compute the embedding of each DNA sequence and perform K-means clustering by setting the num_clusters as the number of species that exist in this dataset. We employ the Adjusted Rand Index (ARI) as the evaluation metric. ARI is a measure of the similarity between two data clusterings, adjusted for chance, providing a normalized index that ranges from −1 to 1. A higher ARI score indicates better performance.

Table 1 shows the models’ performance on clustering. As shown in the table, DNABERT-S consistently achieves the best performance on all the datasets and doubles the performance of the strongest baseline on average. Among all the baselines, TNF and its variant TNF-K achieve the best performance, explaining their wide usage in metagenomics binning. Yet, TNF’s performance is heavily limited since it is not learnable. TNF-VAE represents a naive algorithm that enables learning with TNF, yet it leads to big performance degradation, potentially resulting from the large gap between its training objective and the specific downstream application. Similarly, pre-trained Kmer embeddings from DNA2Vec also fail to effectively cluster different species.

Existing genome foundation models training with language modeling objectives, such as HyenaDNA and DNABERT-2, despite their remarkable performance on labeled datasets, also fail to generate representative embedding without fine-tuning. The phenomenon that pre-trained foundation models underperform descriptive textual features in generating embedding for clustering and retrieval is also observed in the field of natural language processing [Reimers and Gurevych, 2019].

Furthermore, by comparing the DNA-Dropout and DNA-Mutate with DNABERT-2, we found that those popular unsupervised positive pair methods used in contrastive learning in NLP, such as sentence swap/deletion and dropout, do not benefit DNA embedding learning. The DNA-Double, which utilizes the unique double-strain characteristics of DNA sequences, empowers DNABERT-2 to achieve a similar level of performance as TNF. Comparison between DNABERT-S and these variants indicates the importance of appropriate training data construction.

### 5.3 Classification

In this task, we evaluate the embedding quality by how well a linear model can classify different species based on a few labeled embeddings. This evaluation is also known as linear probing. As shown in Table 3, all the datasets we use for classification consist of 100 DNA sequence for each species. We first compute the embedding of each DNA sequence with each model. In each evaluation run, we independently select 80 embeddings from each species to form the test set. For the rest DNA sequences, we respectively sample 1, 2, 5, 10, and 20 embeddings from each species to form the training set. A Logistic Regression model is trained on the training set and evaluated on the test set. We use the macro F1 score as the evaluation metric.

**Figure 3:**
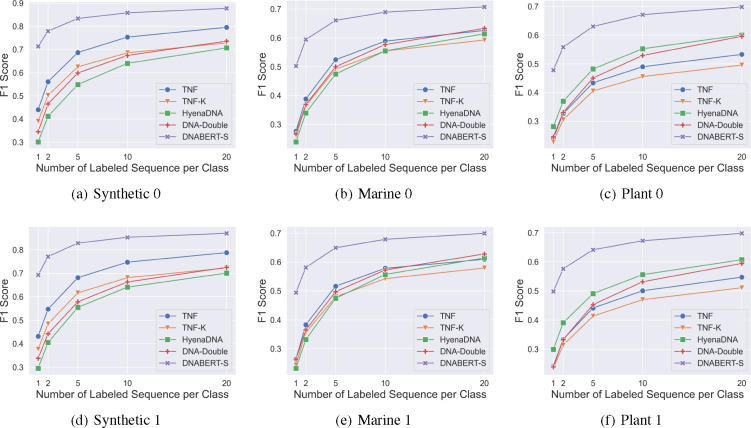
Model’s performance of species classification with varying numbers of training samples on 6 datasets. Results on other 6 datasets are consistent and are presented in Figure 5.

Figure 3 shows the models’ performance on 6 datasets. The remaining results are consistent and are presented in Appendix C.2. As shown in the figure, DNABERT-S consistently achieves the best performance. Remarkably, DNABERT-S achieves better performance than the strongest baseline with only 20% of training data. For example, with only 2 training samples per category, DNABERT-S achieves higher F1 scores than the strongest baseline with 10 training samples. With the same amount of training samples, DNABERT-S significantly outperforms the baselines by a large gap. Notably, in the Synthetic datasets, where none of the species are seen during the contrastive training, a linear model trained with DNABERT-S embeddings achieves an F1 score of over 0.8 in 200 classes classification with only 5 labeled samples in each species, showing DNABERT-S’s capability in generalizing well on unseen data.

### 5.4 Metagenomics Binning

Metagenomics binning is a crucial process in microbial ecology, involving the categorization of DNA sequences into groups that represent individual species. State-of-the-art metagenomics binning method [Kang et al., 2015, 2019; Nissen et al., 2021] always formulate this problem as a clustering problem with an unknown number of clusters based on the feature of each DNA sequence. The DNA sequence feature is often computed by combining sequence-based DNA embedding with various other features and the clustering algorithms are often complicated and strongly correlated with the features they utilize.

In our evaluation, to create a fair environment for DNA embedding benchmarking, instead of relying on any existing tool, we implement the modified K-medoid clustering algorithm proposed in Kang et al. [2015] for metagenomics binning due to its simplicity and effectiveness. Algorithm 1 describes the unsupervised clustering algorithm we used for metagenomics binning, where $s\left(E_{i},E_{j}\right)$ represents the cosine similarity of two vectors $E_{i}$ and $E_{j}$. **Selection of threshold** γ. As shown in Algorithm 1, the threshold γ is the most important hyperparameter that greatly impacts the final binning results. A high threshold results in small and dense clusters while a low threshold results in large yet sparse clusters. Since different models generate embeddings with distinct distributions, a fixed threshold (e.g., 0.9) could be too high for one model yet too low for another one. In practice, massive hyperparameter searches are needed to determine the best threshold for each model on different datasets. Due to the large size of our experiments and the various types of models we used, an automatic way is needed to fairly choose the threshold for each model on each dataset. For each metagenomics binning dataset, we use the dataset from the same source (e.g., Marine) as it with ID 0 to compute a threshold for each model on it, Specifically, we generate embeddings for each DNA sequence in the dataset and compute the similarities between each DNA sequence and its species center (i.e., the average of all the DNA sequence belongs to this species). The 70 percentile of all the similarities is used as the threshold. **Other hyperparameters.** We set minimum bin size m=10, number of steps Z=1000, and number of iterations T=3. We also experimented with T=3,4,5 and 60,70,80,90 percentile of all the similarities is used as the threshold γ, and found that the results are robust to these hyperparameters.

Following Kang et al. [2015, 2019], we formulate this problem as identifying non-overlapping clusters of DNA sequences from the entire dataset, where each cluster of sequence is considered as an identified species. We then compare the predicted clusters with the true labels to count the number of species that have been successfully identified. A species is considered to be successfully identified if the F1 score of this species is over 0.5. We compare different models by the number of species they identify with different levels of F1 scores (e.g., 0.5 – 0.6, 0.8 – 0.9). We only use the DNA embeddings as the feature of each DNA sequence.

**Algorithm 1:** Modified K-Medoid Clustering

1: **Input:** threshold γ, minimum bin size m, embeddings $E\in R^{N\times d}$, number of steps Z, number of iterations T
2: **Initialize:** predictions $p\in R^{N},p_{i}=-1$ for i=1,…,N, similarity matrix $S=EE^{⊤}$ with $S_{ij}=0$ if $S_{ij}<\gamma$, density vector $d\in R^{N}$ with $d_{i}=\sum_{j=1}^{N}\;S_{ij}$
3: **for** step z=1 to Z **do**
4: Select seed index $s={\text{arg max}}_{s^{\prime }}d_{s^{\prime }}$ and corresponding seed $E_{s}$
5: **for** iteration t=1 to T **do**
6: Find neighborhood indices ℐ of $E_{s}$ where $s\left(E_{i},E_{s}\right)>\gamma$ and $p_{i}=-1$ for each i∈ℐ
7: Update seed: $E_{s}-\frac{1}{\left|ℐ\right|}\sum_{i\in ℐ}\;E_{i}$
8: **end for**
9: Set $p_{i}\leftarrow z,d_{i}\leftarrow 0$ for each i∈ℐ
10: Set $d_{x}+d_{x}-\sum_{i\in ℐ}\;S_{xi}$ for each x∈[1,2,…,N]
11: **end for**
12: **for** step z=1 to Z **do**
13: Find indices ℐ where $p_{1}=z$ for each i∈I
14: **if** |I|<m **then**
15: Set $p_{i}\leftarrow -1$ for each i∈ℐ
16: **end if**
17: **end for**
**Return:** predictions p

**Figure 4:**
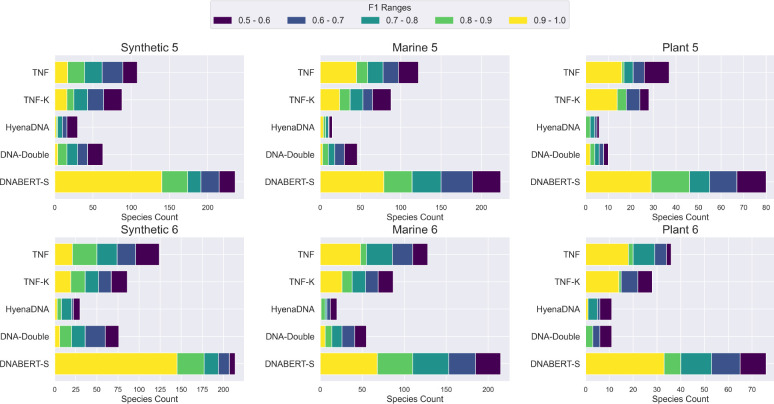
Metagenomics Binning Results. The bin size represents the number of unique species identified by each model and different colors represent the F1 score of the identified species. With high F1 scores, DNABERT-S identifies many more species than the baselines.

Figure 4 shows the models’ performance on 6 metagenomics binning datasets. As shown in the figure, similar to our observation in clustering, DNABERT-S identifies twice the number of species with an F1 score of over 0.5 compared to the strongest baseline, showing its great capability in tackling important real-world biology challenges. Notably, DNABERT-S identifies a large number of species with an F1 score over 0.9. indicating its capability to accurately segregate different species in the embedding space, aligning with our observation in Figure 1. In the Synthetic datasets, where the sequences are error-less (extracted from reference genome) and the number of sequences in each species is more balanced, DNABERT-S recovers over 80% of the species with an F1 score of over 0.5 purely based on the DNA sequences themselves. In more realistic datasets such as Marine and Plant, where noise (e.g., error from sequences) exists in DNA sequence and species size is highly imbalanced, DNABERT-S is still able to recover about 40% of the species with an F1 score of over 0.5.

### 5.5 Ablation Study

In this section, we present our ablation studies on DNABERT-S. We perform the ablation study on CAMI2 datasets with both clustering and classification. To validate the effectiveness of curriculum learning, we compare DNABERT-S with three of its variants, each of which is trained purely with the Weight SimCLR [Zhang et al., 2021], i-Mix [Lee et al., 2020], and our proposed Manifold Instance Mixup (MI-Mix) loss. To examine the effectiveness of MI-Mix, we also compare it with a variant trained with the curriculum contrastive method that replaces MI-Mix with i-Mix in the second phase. All the variants are trained with the same data and hyperparameters.

As shown in Table 2, our curriculum learning strategy that combines Weighted SimCLR and MI-Mix achieves the best performance. Our method outperforms both variants that are trained purely with Weighted SimCLR and MI-Mix loss, showing the effectiveness of our proposed curriculum contrastive learning strategy. Moreover, the comparison among the three variants that are trained with a single loss function indicates the effectiveness of MI-Mix in learning DNA embeddings.

## 6 Conclusion

We introduced DNABERT-S, a novel genome foundation model designed to generate effective, species-aware DNA embeddings. To facilitate the training of DNABERT-S, we introduce the Manifold Instance Mixup (MI-Mix) training objective and the Curriculum Contrastive Learning (C^2^LR) strategy. We perform extensive experiments on 18 datasets across a variety of challenging tasks, including species clustering, classification, and metagenomics binning, to demonstrate the remarkable ability of DNABERT-S.

### Limitations and Broader Impact.

We expect the DNABERT-S to greatly benefit a wide range of species-related research, including but not limited to species identification, metagenomics binning, biodiversity assessment, and understanding of evolutionary relationships. Despite its specialized focus on species-aware tasks, as noted in Appendix C.6, DNABERT-S’s species-aware training approach does not inherently enhance its performance in unrelated genomic tasks, such as promoter prediction in the human genome. Nevertheless, the methodologies and insights gleaned from our extensive empirical analyses offer valuable pathways for advancing more detailed and accurate investigations across a wide spectrum of genomic applications.

## Figures and Tables

**Table 1:** Models’ performance on K-Means clustering measured by Adjusted Rand Index (ARI). DNABERT-S doubles the ARI of the strongest baseline on average.

Model	Synthetic		Marine					Plant					Ave.
	0	1	0	1	2	3	4	0	1	2	3	4	

**TNF**	38.75	37.76	25.65	25.31	26.05	20.67	23.47	25.80	24.23	24.81	22.72	22.39	26.47
**TNF-K**	36.26	35.66	25.99	25.00	26.27	21.15	23.27	25.60	25.58	26.45	22.59	21.76	26.30
**TNF-VAE**	25.94	24.60	16.28	16.52	16.27	12.92	15.02	18.40	16.51	17.53	14.08	14.38	17.37
**DNA2Vec**	24.68	23.34	16.07	15.99	16.18	12.62	14.51	20.13	19.77	20.25	17.24	16.37	18.10
**HyenaDNA**	20.04	18.99	16.54	16.64	16.47	13.35	14.85	24.06	25.33	26.18	21.01	21.16	19.55
**NT**	8.69	9.63	4.92	4.74	5.02	3.68	4.31	7.00	6.32	6.37	5.54	5.42	5.97
**DNABERT-2**	15.73	16.74	13.24	13.53	12.99	10.41	11.87	15.70	16.28	16.32	13.99	13.66	14.21

**DNA-Dropout**	16.64	16.08	11.89	11.77	11.89	9.85	10.31	16.18	15.41	16.95	13.53	13.85	13.70
**DNA-Double**	35.11	34.14	27.05	27.23	26.56	21.47	24.39	22.35	21.35	23.03	19.44	19.06	25.10
**DNA-Mutate**	16.55	16.24	11.40	11.53	11.34	9.03	10.02	14.27	14.13	16.22	12.01	11.68	12.87

**DNABERT-S**	**68.21**	**66.33**	**53.98**	**52.56**	**51.99**	**46.39**	**50.49**	**51.43**	**51.56**	**51.11**	**50.44**	**51.15**	**53.80**

**Table 2:** Ablation study on the Curriculum Contrastive Learning (C^2^LR) and Manifold Instance Mixup (MI-Mix).

Training Objective	Clustering	Classification

W. SimCLR + MI-Mix (ours)	51.11	60.88
W. SimCLR + i-Mix	−3.46	−3.56
only W. SimCLR	−1.13	−1.17
only MI-Mix	−0.66	−0.42
only i-Mix	−5.25	−4.76

**Figure 5:**
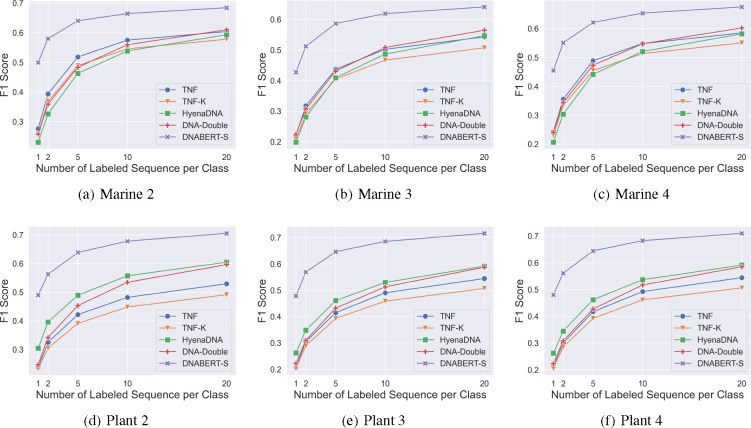
Results of species classification on other 6 datasets.

**Figure 6:**
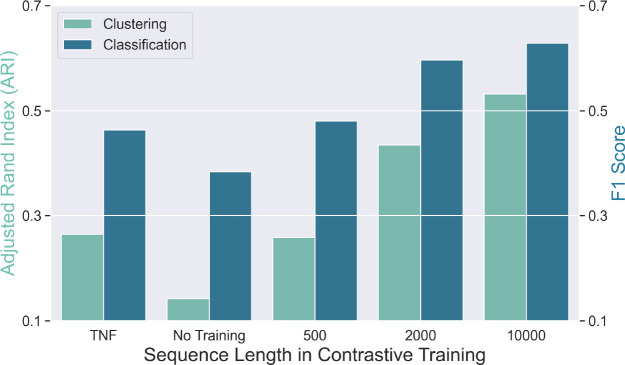
Performance of DNABERT-S and TNF on clustering (upper) and classification (lower) with different input sequence lengths during evaluation.

**Figure 7:**
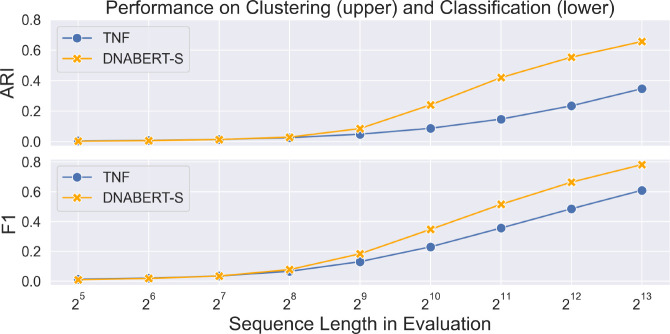
Performance of DNABERT-S and TNF on clustering (upper) and classification (lower) with different input sequence lengths during evaluation.

**Figure 8:**
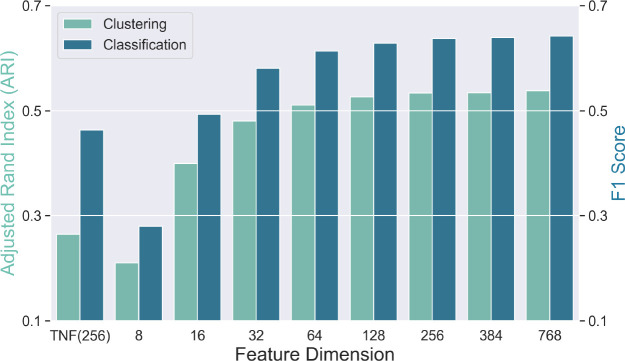
DNABERT-S’s performance with varying embedding dimensions reduced by average pooling. DNABERT-S is robust to feature dimension reduction, and it even outperforms TNF with 16-dimensional embedding.

## A i-Mix for Original Contrastive Learning

The Weighted SimCLR method treat each instance ${\left\{x_{i}\right\}}_{i=1}^{B}$ and ${\left\{x_{i^{+}}\right\}}_{i=1}^{B}$ as anchors, with each anchor associated with 2B–2 negative samples. This section explains why integrating the i-Mix method into Weighted SimCLR (Sec. 3.1) doubles memory or training time.

For clarity, we define $x_{B+i}=x_{i^{+}}$. We also expand the virtual labels $v_{i}$, which are 2B-dimensional, to identify the positive sample for each anchor. Here $v_{i,\tilde{i}}=1$ and $v_{i,j\neq \tilde{i}}=0,$ $\tilde{i}=\left(B+i\right)\;mod\;2B$.

When the i-Mix method treats every sample from ${\left\{x_{i}\right\}}_{i=1}^{2B}$ as anchor, it begins by shuffling ${\left\{\left(x_{i},v_{i}\right)\right\}}_{i=1}^{2B}$ to generate ${\left\{\left({\overset{ˆ}{x}}_{i},{\overset{ˆ}{v}}_{i}\right)\right\}}_{i=1}^{2B}$. For each anchor $\left(x_{i},v_{i}\right)$, it uses a simple mixup method to mix it up with $\left({\overset{ˆ}{x}}_{i},{\overset{ˆ}{v}}_{i}\right)$ before encoder layers of the model, using mixup coefficient $\lambda_{i}∼Beta(\alpha ,\alpha )$. This mixing results in:

$$\left(h_{i}^{0},v_{i}^{mix}\right)=\left(\lambda_{i}x_{i}+\left(1-\lambda_{i}\right){\overset{ˆ}{x}}_{i},\lambda_{i}v_{i}+\left(1-\lambda_{i}\right){\overset{ˆ}{v}}_{i}\right).$$

Moreover, for each anchor $x_{i}$, if i-Mix considers all samples from ${\left\{x_{j}\right\}}_{j=1}^{2B}\backslash \left\{x_{i}\right\}$ as either positive or negative samples, the model f(⋅) must process both the initial data instances ${\left\{x_{i}\right\}}_{i=1}^{2B}$ and mixed data instances ${\left\{h_{i}^{0}\right\}}_{i=1}^{2B}$ to generate their embeddings. Therefore, the i-Mix requires nearly twice more memory or training time compared to the method in Sec. 3.1 if using the same batch size.

## B Data Statistics of Evaluation Benchmark

This section details the comprehensive statistics of the 18 datasets utilized for evaluating various DNA embedding models, as summarized in Table 3. For tasks involving clustering and classification, each dataset encompasses between 93 to 499 distinct species. From each species, 100 DNA sequences are sampled. These sequences vary in length, ranging from 2,000 to 20,000 base pairs. In the case of metagenomics binning, the datasets exhibit an unbalanced distribution of sequences across different species. Specifically, the number of sequences per species varies significantly, ranging from as few as 10 to as many as 4,599.

**Table 3:** Data statistics of the datasets for the DNA embedding evaluation. This table presents the sampling source, ID, number of sequences, number of sequences, and the minimum / maximum / medium values of the sequence lengths and number of sequences in each species. We use the same set of balanced datasets for clustering and classification and another set of datasets for metagenomics binning.

Tasks	Source	ID	Species	Sequences	Sequence Length	Sequences Per Species

**Clustering & Classification**	Synthetic	0	200	20000	10k / 10k / 10k	100 / 100 / 100
	Synthetic	1	200	20000	10k / 10k / 10k	100 / 100 / 100
	Marine	0	326	32600	2k / 20k / 7.6k	100 / 100 / 100
	Marine	1	375	37500	2k / 20k / 8.2k	100 / 100 / 100
	Marine	2	361	36100	2k / 20k / 8.5k	100 / 100 / 100
	Marine	3	499	49900	2k / 20k / 6.8k	100 / 100 / 100
	Marine	4	360	36000	2k / 20k / 7.1k	100 / 100 / 100
	Plant	0	108	10800	2k / 20k / 6.6k	100 / 100 / 100
	Plant	1	100	10000	2k / 20k / 6.4k	100 / 100 / 100
	Plant	2	93	9300	2k / 20k / 6.2k	100 / 100 / 100
	Plant	3	129	12900	2k / 20k / 5.5k	100 / 100 / 100
	Plant	4	129	12900	2k / 20k / 5.7k	100 / 100 / 100

**Metagenomics Binning**	Synthetic	5	323	37278	10k / 10k / 10k	31 / 200 / 111
	Synthetic	6	249	28206	10k / 10k / 10k	30 / 199 / 114
	Marine	5	515	119465	2.5k / 20k / 4.3k	10 / 841 / 201
	Marine	6	527	125194	2.5k / 20k / 4.4k	10 / 915 / 223
	Plant	5	181	71642	2.5k / 20k / 3.7k	10 / 4293 / 190
	Plant	6	196	68426	2.5k / 20k / 3.7k	10 / 4599 / 116

## C More Experimental Results

### C.1 Comparison Between Different Foundation Model

This subsection delineates a comparative analysis of various existing genome foundation models in the context of DNA embedding generation. We evaluated four renowned models: DNABERT [Ji et al., 2021], DNABERT-2 [Zhou et al., 2023], Nucleotide Transformer [Dalla-Torre et al., 2023], and HyenaDNA [Nguyen et al., 2023]. Notably, DNABERT and the Nucleotide Transformer exhibit strict input sequence length limitations of 512 and 6144 (V1) or 12288 (V2) base pairs, respectively. Conversely, DNABERT-2 and HyenaDNA do not impose such constraints. Considering the potentially extensive length of genome sequences in metagenomics binning, our preliminary experiments focused solely on DNABERT-2 and HyenaDNA.

We train both models on our pre-training datasets with the same set of hyperparameters for 3 epoch. We save checkpoints periodically and select the best checkpoint based on the models’ validation loss on the validation set. Since HyenaDNA, in general, requires larger learning rates than DNABERT-2, we train it with three different learning rates (3e-4,3e-5, and 3e-6) and select the one that works best. For DNABERT-2, we only train it once with a learning rate of 3e-6. To avoid the impact of other factors such as the schedule of curriculum learning, we train both models with the Weighted SimCLR loss only in the entire training process. We then evaluate the models before and after contrastive training on our evaluation benchmark.

Table 4 presents the performance of both pre-trained DNABERT-2 and HyenaDNA, with and without contrastive training, in K-means clustering evaluations. DNABERT-2, despite underperforming without contrastive training, demonstrated superior performance compared to HyenaDNA post-training, highlighting its efficacy in learning effective DNA embeddings. Similar trends are observed in few-shot species classification scenarios, as detailed in Table 5. While DNABERT-2 initially exhibited subpar performance, it significantly improved post-contrastive training, achieving the best results. Thus, DNABERT-2 was chosen as the foundational model for DNABERT-S.

**Table 4:** Performance of DNABERT-2 and HyenaDNA on K-Means clustering measured by Adjusted Rand Index (ARI). Here, ∆ represents the model’s performance improvement after contrastive training,

Dataset ID	Synthetic		Marine					Plant					Ave.
	0	1	0	1	2	3	4	0	1	2	3	4	

**DNABERT-2 w/o**	15.73	16.74	13.24	13.53	12.99	10.41	11.87	15.70	16.28	16.32	13.99	13.66	14.21
**DNABERT-2 w/**	**69.33**	**68.37**	**53.18**	**51.94**	**51.91**	**46.60**	**49.69**	**49.05**	**50.33**	**49.68**	**48.57**	**49.83**	**50.08**
**∆**	53.61	51.63	39.94	38.41	38.93	36.19	37.82	33.35	34.05	33.36	34.57	36.17	39.00

**HyenaDNA w/o**	20.04	18.99	16.54	16.64	16.47	13.35	14.85	24.06	25.33	26.18	21.01	21.16	19.55
**HyenaDNA w/**	57.58	55.19	42.92	42.68	42.24	36.17	40.10	41.42	40.36	40.46	36.87	38.25	42.85
**∆**	37.54	36.20	26.38	26.04	25.77	22.82	25.24	17.36	15.03	14.28	15.86	17.09	23.30

**Table 5:** Performance of DNABERT-2 and HyenaDNA on few-shot classification measured by Macro F1 score. Here, ∆ represents the model’s performance improvement after contrastive training,

Num Shots	Synthetic				Marine				Plant				Ave.
	1	2	5	10	1	2	5	10	1	2	5	10	

**DNABERT-2 w/o**	24.43	34.81	48.93	58.58	19.50	28.45	40.64	48.98	21.04	28.16	38.50	45.46	36.46
**DNABERT-2 w/**	**72.02**	**78.63**	**84.55**	**86.99**	**48.23**	**57.90**	**64.80**	**67.94**	**44.97**	**52.35**	**60.35**	**64.63**	**65.28**
**∆**	47.60	43.83	35.62	28.41	28.73	29.45	24.16	18.96	23.92	24.20	21.85	19.17	28.82

**HyenaDNA w/o**	30.13	41.18	54.86	64.03	23.92	33.94	47.47	55.50	28.15	36.97	48.20	55.24	43.30
**HyenaDNA w/**	59.58	67.79	74.62	78.53	43.60	53.70	62.00	65.55	43.46	52.12	59.40	62.80	60.26
**∆**	29.45	26.62	19.76	14.50	19.68	19.76	14.52	10.05	15.31	15.15	11.20	7.55	16.96

### C.2 Remaining Results on Species Classification

In this section, we present the models’ performance on species classification in the other 6 datasets that are not presented in Section 5.3 due to space limits. As shown in Figure 5, the results are consistent with those shown in Figure 3.

### C.3 Impact of Sequence Length

This section delves into the influence of DNA sequence length on final model performance, examined from both training and evaluation standpoints.

#### C.3.1 Varying Sequence Length in Training

Training with longer DNA sequences increases the need for more memory and computing power. It also means we can only use smaller batches of data at a time. Therefore, the length of the sequences is an important factor in contrastive training as it affects how much it costs to train the model. To see how different sequence lengths affect training, we did three experiments using the same data. Our training data has sequences that are 10000bp long. For experiments with shorter sequences S, we only used the first S nucleotides of each DNA sequence. We tested sequence lengths of 500bp, 2000bp, and 10000bp, training only with Weighted SimCLR loss and starting from the pre-trained DNABERT-2 model.

Figure 6 shows the results for the three models, along with the pre-trained DNABERT-2 without contrastive training and the strongest baseline, TNF. The findings reveal that sequence length significantly influences the model’s performance. Training even on short sequences, such as 500bp, leads to substantial improvements. The model trained with 500bp sequences performs nearly as well as TNF. When we increase the input sequence length from 500bp to 2000bp, there’s a marked improvement in performance. A similar trend is observed when increasing the sequence length from 2000bp to 10000bp. These results highlight the importance of sequence length in training an effective model. Therefore, we decided to train our model with 10000bp sequences, despite the higher computational requirements.

#### C.3.2 Varying Sequence Length in Evaluation

In this part, we assess how the length of DNA sequences in evaluation impacts performance. We use two synthetic datasets for clustering and classification tasks. Each sequence in these datasets is deliberately constructed to be 10000bp long. This allows us to create a test set where all sequences have the same length. We test sequence lengths ranging from 32bp (2^5^) to 8192bp (2^13^). For each test with different sequence lengths, we keep everything else the same, like how we split the data into training and testing sets and the settings for logistic regression.

Figure 7 presents the performance of TNF and DNABERT-S with various sequence lengths. The results show that both models significantly benefit from longer sequences. When the sequence length is less than 256bp (2^8^), both models perform poorly in clustering and classifying samples. However, as the sequence length increases, starting from 512bp (2^9^), DNABERT-S begins to outperform TNF. The performance gap between the two models gets bigger as the sequence length increases. These findings highlight the crucial role of sequence length in effectively differentiating between species.

### C.4 Impact of Embedding Dimension Reduction

This section investigates how changes in embedding dimensions affect the performance of DNABERT-S, a key aspect influencing the scalability of DNA embeddings generated by the model. Initially, DNA embeddings for all clustering and classification datasets are computed using the pre-trained DNABERT-S. To reduce embedding dimensions, we use an average pooling layer with a consistent kernel size and stride S. This process effectively averages S consecutive dimensions into one new dimension. We test with S values of 96, 48, 24, 12, 6, 3, and 2, corresponding to reduced embedding dimensions of 8, 16, 32, 64, 128, 256, and 384, respectively.

Figure 8 illustrates DNABERT-S’s performance with these varying feature dimensions, in comparison to TNF. The results demonstrate that DNABERT-S’s embedding is quite resilient to dimension compression. It maintains nearly the same performance level even when reduced to 256 dimensions and only experiences a notable drop in performance when compressed to 32 dimensions. Remarkably, DNABERT-S still surpasses the 256-dimensional TNF feature even when its own dimensionality is reduced to just 16. This robustness to dimension reduction enhances its practical applicability in various genomic contexts.

**Table 6:** Experimental results on the fungi and species classification dataset on GUE [Zhou et al., 2023]. DNABERT-S still outperforms DNABERT-2 when labeled data is abundant.

Model	Fungi	Viral

DNABERT-2	91.37	35.44
DNABERT-S	**94.64**	**41.91**

### C.5 Results on Scenario with Abundant Labeled Data

While previous experiments have highlighted DNABERT-S’s exceptional performance in scenarios with limited or no labeled data, this section focuses on its effectiveness in situations where abundant labeled data is available.

We utilize the fungi and viral species classification datasets from Zhou et al. [2023]. The fungi dataset contains 20 different species, each with 400 training samples that are 10,000 base pairs (10000bp) long. The viral dataset comprises 40 different species, with each species providing 100 training samples of 5,000 base pairs (5000bp) in length. Similar to our approach in the few-shot classification evaluation, we use DNABERT-2 and DNABERT-S to generate embeddings for each DNA sequence and subsequently train a logistic regression model for classification purposes. In alignment with Zhou et al. [2023], we employ the Matthews Correlation Coefficient (MCC) as our evaluation metric.

Table 6 presents the performance comparison between DNABERT-2 and DNABERT-S. As indicated by the results, DNABERT-S continues to demonstrate significant performance improvements even in the presence of a large volume of training data. This suggests that DNABERT-S’s capabilities are not confined to scenarios with scarce data but also extend effectively to data-rich environments, reinforcing its versatility and robustness in various genomic analysis contexts.

### C.6 Results on Other Types of Tasks

This section evaluates DNABERT-S’s performance across a range of distinct genomic tasks. We utilize the GUE benchmark [Zhou et al., 2023], which comprises a comprehensive collection of 28 datasets covering 7 diverse tasks, such as epigenetic marks prediction, promoter prediction, and transcription factor binding site prediction. Following our established methodology, DNABERT-2 and DNABERT-S are used to generate embeddings for each DNA sequence, and a logistic regression model is trained for classification. The Matthews Correlation Coefficient (MCC) serves as the evaluation metric.

**Table 7:** Performance of DNABERT-2 and DNABERT-S on the GUE benchmark.

	Epigenetic Marks Prediction							
	H3	H3K14ac		H3K36me3		H3K4me1	H3K4me2	H3K4me3

**DNABERT-2**	66.87	**38.92**		**43.39**		31.79	**30.16**	**23.68**
**DNABERT-S**	69.02	37.45		41.91		32.76	27.86	22.16

	Epigenetic Marks Prediction					Promoter Detection		
	H3K79me3		H3K9ac	H4	H4ac	all	notata	tata
**DNABERT-2**	57.01		**47.52**	73.75	35.54	78.31	40.25	**88.85**
**DNABERT-S**	**58.41**		44.70	**75.96**	**35.64**	**78.93**	**40.59**	88.62

	Transcription Factor Prediction (Human)					Core Promoter Detection		
	0	1	2	3	4	all	notata	tata

**DNABERT-2**	**62.67**	**68.58**	**54.44**	**35.67**	**62.89**	**57.33**	**46.18**	61.48
**DNABERT-S**	60.34	65.28	47.75	30.54	59.45	56.52	39.05	**61.87**

	Transcription Factor Prediction (Mouse)					Virus	Splice	
	0	1	2	3	4	Covid	Reconstruct	Ave.

**DNABERT-2**	**37.08**	69.56	**67.71**	**42.26**	**34.80**	**54.50**	**24.85**	**51.28**
**DNABERT-S**	31.38	**71.13**	56.71	39.85	28.94	49.84	23.87	49.16

The results, as detailed in Table 7, show that DNABERT-2 outperforms DNABERT-S on 19 of the 28 datasets, with an average improvement of 2.12 in the MCC. This outcome aligns with expectations, given the distinct objectives of DNABERT-S and the GUE benchmark. DNABERT-S is specifically designed to generate species-aware embeddings, which are optimized for clustering and differentiating various species. In contrast, the tasks in the GUE benchmark, which predominantly involve predicting functional aspects of human and mouse genomes, require a different set of embedding characteristics. Therefore, it’s evident that DNABERT-S, while not universally superior in all aspects of DNA embedding generation, excels in its targeted domain. The training approach and methodologies developed for DNABERT-S, despite the model’s specialized focus, hold potential applicability across various genomic analysis scenarios
